# Factors Associated With Depressive Symptoms Among Graduate Students in Science and Technology: A Cross‐Sectional Study With Multivariable Analysis

**DOI:** 10.1155/da/8886228

**Published:** 2025-12-17

**Authors:** Saphal Chapagai, Ishwari Prasad Banjade, Shankar Prasad Khanal

**Affiliations:** ^1^ Central Department of Statistics, Institute of Science and Technology, Tribhuvan University, Kirtipur, Kathmandu, Nepal, tribhuvan-university.edu.np

**Keywords:** depressive symptoms, graduate students, multivariable logistic regression

## Abstract

**Background:**

Depression among graduate students is a growing concern, according to the WHO. However, existing literature lacks multivariable analysis focused specifically on the graduate student populations. This study addresses this gap by investigating the factors contributing to depressive symptoms among science and technology graduate students.

**Methods:**

A cross‐sectional study was conducted after ethics committee approval among 387 consenting graduate students selected using stratified random sampling from all 13 departments at the Institute of Science and Technology (IOST), Tribhuvan University (TU), Nepal. The data were collected via a self‐administered questionnaire, verifying the outcome variable depression by the Patient Health Questionnaire‐9 (PHQ‐9) scale (no/low depressive symptoms: 0–9 points; moderate/severe depressive symptoms: 10–27 points). Multivariable logistic regression was used to identify factors associated with moderate/severe depressive symptoms, calculating the adjusted odds ratio (OR) with 95% confidence interval (CI).

**Results:**

The prevalence of moderate/severe depressive symptoms among graduate students was 23% (95% CI: 19–27). Statistically significant (*p*  < 0.05) associated factors were financial stress (OR: 2.35, 95% CI: 1.30–4.26), relationship dissolution (OR: 2.22, 95% CI: 1.06–4.63), stressful assignments (OR: 5.09, 95% CI: 2.31–11.26), medium self‐esteem (OR: 4.73, 95% CI: 2.43–9.18), and low self‐esteem (OR: 10.21, 95% CI: 4.71–22.11).

**Conclusion:**

The unique contributors to depressive symptoms identified in this study through multivariable analysis, adjusting for confounding, emphasize the need for targeted mental health support among science and technology graduate students.

## 1. Introduction

Depression, a common mental health disorder in which people feel sad or lose interest in activities [1], is estimated to affect 3.8% of the global population, that is, ~280 million people [2]. The prevalence of depressive symptoms among graduate students is about one in three of the student population across different regions [3]. Moreover, this prevalence among university students has remained largely unchanged over the past decades [4], highlighting the importance of identifying its associated factors. Some factors have been found to be associated with depressive symptoms globally among graduate students. For instance, a study in Turkey found that younger age, being single, pursuing a master’s degree, and seeking psychological assistance were linked to depressive symptoms [5]. This study only presented crude results without adjustment for confounding. More research is being done in an undergraduate setting. For example, among Chinese university students, study years, age, parental relationships, satisfaction with major, mother’s education, and family income status were identified as significant variables [6]. In Saudi Arabia, factors such as the loss of family members, difficulties in personal relationships, and failing an academic year were also identified as key contributors to depressive symptoms [7]. In Nepal, risk factors such as age, ethnicity, faculty of study, academic pressure, substance use, sexual activity, and lack of physical exercise have been identified among undergraduate students [8–11]. However, studies specifically examining depressive symptoms among science and technology graduate students remain scarce in Nepal, even though these students face unique academic and research pressures that remain unexplored in studies of undergraduates.

Graduate students face different challenges that have not been adequately represented in the literature. Compared with their undergraduate peers, they commonly faced competitive academic environments, financial difficulties, extensive research work, and uncertainty about their future careers [12, 13]. These challenges increase the risk of experiencing adverse mental health problems, such as depressive symptoms. Students pursuing a graduate degree in science and technology face additional challenges like rigorous academic coursework, a significant time commitment to lab work and paper preparation for publication, and a lack of mental health support [13, 14]. These factors might enhance the risk of depressive symptoms compared to other students in nonscience and technology fields. Furthermore, graduate students have a different academic context and psychosocial stressors than undergraduates, who have been the main subject of nearly all previous research in Nepal. However, multivariable analysis research on science and technology graduate students in Nepal is lacking. This leaves a critical gap in understanding the factors associated with depressive symptoms among these students.

To address this existing gap, this study aims to determine the prevalence of depressive symptoms and identify associated risk factors among graduate students by examining sociodemographic, socioeconomic, personal, behavioral, and psychological well‐being variables using multivariable logistic analyses.

## 2. Methods

This study was conducted according to the recommended observational design and reported following the Strobe checklist [15].

### 2.1. Study Design and Ethical Approval

A cross‐sectional study was carried out between February 13 and August 12, 2024. The study obtained all required ethical approval from the Institutional Review Committee of the Institute of Science and Technology (IOST), Tribhuvan University (TU) (Regd. No IRCIOST‐24‐0001). The students were required to provide written informed consent to participate in the study.

### 2.2. Setting and Participants

This study was based on MSc students from all 13 Central Departments of the University Campus, TU, during the 2021/22 to 2023/2024 academic session. At the IOST, master’s programs are structured into four semesters over 2 years. A total of 1235 students enrolled in the second and fourth semesters across all 13 departments, which included Physics, Chemistry, Mathematics, Statistics, Geology, Environmental Science, Botany, Zoology, Microbiology, Meteorology, Computer Science and Information Technology (CSIT), Biotechnology, and Food Technology. Enrollment lists were obtained from respective departmental administrations. Eligible participants were all master’s level students enrolled in the second or fourth semester of these departments at IOST who provided written informed consent. Students on long‐term leave or those who refused to participate were excluded.

### 2.3. Variables

The primary outcome variable was depressive symptoms, measured using the Patient Health Questionnaire‐9 (PHQ‐9). Explanatory variables included sociodemographic, behavioral, academic, and psychological well‐being factors.

#### 2.3.1. Outcome Variables

Depressive symptoms were measured using the PHQ‐9, a widely validated instrument designed to assess the frequency and severity of depression symptoms over the past 2 weeks. Each of the nine items is rated on a scale from 0 (*not at all*) to 3 (*nearly every day*), yielding a composite score ranging from 0 to 27. Based on the total score, depressive symptoms were categorized into no/low depressive symptoms (0–9 points) and moderate/severe depressive symptoms (10–27 points). A PHQ‐9 score of ≥10 demonstrated a sensitivity of 88% and specificity of 88% for detecting moderate/severe depressive symptoms [16]. The internal consistency of the PHQ‐9 in this study, measured by Cronbach’s alpha, was 0.824.

#### 2.3.2. Sociodemographic and Socioeconomic Variables

These variables included gender (male/female), ethnicity (Brahmin/Chettri, Madhesi, Adiwasi/Janjati, Dalit), family monthly income (categorized as, less than Rs 25,000; 25,000–50,000; 50,000 and above), financial stress ( yes/no), current living residence (hostel, with family, alone, with a friend), choice of study (own choice or family pressure), marital status (single, married, divorced, living together), chronic disease in a family member (yes/no), and parental relationship (poor, fair, good) based on the respondent’s perceptions. For the current living residence variable, “hostel” referred to on‐campus student accommodation provided by the university, whereas the other categories indicated private living arrangements outside the campus.

#### 2.3.3. Personal and Behavioral Variables

These variables included smoking habit (never, present, past, occasionally), alcohol intake habit (never, present, past, occasionally), death of a family member (parent, sibling, or spouse) within past year (yes/no), relationship dissolution (yes/no), worry about the future (no/slightly, moderately, extremely), and sleeping hours (categorized into “<6 hours”, “between 6 and 8” hours, “> 8 hours”). For both smoking and alcohol intake, “occasionally” referred to infrequent use during social gatherings and festivals, whereas “present” indicated more consistent use.

#### 2.3.4. Academic Variables

These variables included manageable course load, adequate support from a subject‐specific teacher, positive relationship with the teacher, high academic pressure, enjoyment while working in the lab, and stressful assignments, which are different aspects of the academic variables and were initially measured on a 5‐point Likert scale (1 = *strongly disagree*, 5 = *strongly agree*). The data were then recoded into three categories (disagree, neutral, and agree) for analysis purposes due to low frequency in the extreme categories (strongly disagree and strongly agree).

#### 2.3.5. Psychological Well‐Being Variables

Perceived social support (PSS) was measured using the Oslo Social Support Scale‐3 (OSSS‐3), which is divided into three categories: weak support (3–8), moderate support (9–11), and strong support (12–14). The Cronbach’s alpha for the PSS in this study was 0.582. However, this value is relatively low due to the brevity of scale. Normally, brief scales have lower alpha values as Cronbach’s alpha depends on the number of items [17].

The Rosenberg Self‐Esteem Scale (RSES) assessed how participants felt about themselves using 10 items, half stating positive things and the other half stating negative things. Before analyzing the results, we reversed the scores for the negative questions. The scale uses a 4‐point response system (1 = *strongly disagree*, 2 = *disagree*, 3 = *agree*, 4 = *strongly agree*), with total scores ranging from 10 to 40. Based on this score, the respondents were divided into three groups: high self‐esteem (≥30 points), medium self‐esteem (26–29 points), and low self‐esteem (≤25 points) [18]. The Cronbach’s alpha coefficient was 0.821 for this study.

### 2.4. Sample Size

A stratified random sampling technique was used to collect the data, considering each of the 13 central departments as a stratum. The sample size was calculated using the formula: *n* = $\frac{z^{2}*p\left(1-p\right)}{e^{2}}$ which resulted in ~384 participants, where *e* = the margin of error (5%), *z* is the standard value of the Z test for the 95% confidence interval (CI), and *p* is the presumed prevalence rate of depressive symptoms, set at 50% in the absence of prior data specific to our study population. Once the total sample size was determined, it was proportionately allocated across strata based on the population distribution. Respondents from each stratum were randomly selected using student lists obtained from respective departments, ensuring diverse representation across various disciplines.

A 5% nonresponse rate was assumed during the study design, resulting in a total sample size of 403 respondents. However, 16 (4.2%) respondents did not participate during the data collection period. Therefore, the final analysis was conducted on 387 respondents, representing 31.1% of the total graduate student population (1235) within the IOST at TU.

### 2.5. Statistical Analysis

The data collected in this study were entered in IBM SPSS Statistics 25.0 and analyzed using descriptive and inferential statistics. Categorical variables were summarized as frequencies and percentages, while continuous variables were presented as mean ± standard error (SE), median, minimum, and maximum values. Bivariate analysis was conducted using the chi‐square test or Fisher’s exact test, based on sample size. Variables with *p*‐values less than 0.05 were included in the multivariate binary logistic regression model through stepwise forward selection. Model fit was assessed using the Hosmer–Lemeshow test, with a *p*‐value >0.05 indicating a good fit. The area under the receiver operating characteristic curve (AUC) was used to evaluate the model’s discrimination ability.

## 3. Results

Based on a sample of 387 respondents, the mean age of the respondents was 25.22 ± 0.084 years, ranging from 22 to 32 years. The majority of respondents, 224 (57.9%), were males, followed by 163 (42.1%) females. Most of the respondents, 283 (73.1%), were Brahmin/Chhetri. Regarding current living residents, 34.9% resided with their families. A total of 330 (85.3%) respondents were single, and 174 (45%) respondents had incomes within the range of 25–50 1000 rupees per month. Financial stress was prevalent among 224 (57.9%) respondents. In terms of the choice of study, 5.4% attributed it to family pressure, while the majority (94.6%) based their decision on self‐preference. Additionally, 303 (78.3%) reported having a good relationship with their parents. Concerning health, 83 (16.3%) respondents indicated having diseases in their family. Most students had no smoking and alcohol consumption habits. A total of 73 (18.9%) reported recent experience with a family member’s death, and 58 (15%) respondents reported breakups with loved ones. Moreover, 29.5% of the students felt moderately worried. Regarding sleeping patterns, 68.5% of the participants slept 6–8 h per day. Regarding the manageability of the study load, 43.4% of the participants agreed that it was manageable. Similarly, 53.5% of respondents felt that they received adequate support from their subject‐specific teachers, 58.9% of participants reported a positive relationship with teachers, 29.5% of respondents agreed that there was academic pressure, 62.0% perceived working in a laboratory as interesting, and 39.8% agreed that assignments were stressful. In terms of self‐esteem and PSS, 53.0% had high self‐esteem, and 49.9% had moderate social support.

### 3.1. Associations Between Sociodemographic, Socioeconomic, Personal and Behavioral, Academic, and Psychological Well‐Being Variables

The respondent’s gender, current living arrangements, financial stress, chronic disease in the family, death of a family member, relationship dissolution, worries about the future, manageable course load, adequate academic support, high academic pressure, stressful assignments, PSS, and self‐esteem levels (*p* − value < 0.05) were significantly associated with depressive symptoms as shown in Tables 1, 2, 3

**Table 1:** Associations of sociodemographic and socioeconomic variables with depressive symptoms.

Variables	*n* (%)	Depressive symptoms		(*p*‐Value)
		No/low	Moderate/severe	
Age (mean ± SE)	(25.23 ± 0.014)	—	—	(0.56^b^)
Gender				
Male	224 (57.9%)	184 (82.1%)	40 (17.9%)	8.685 (0.003)
Female	163 (42.1%)	113 (69.3%)	50 (30.7%)	
Ethnicity				
Brahmin/Chhetri	283 (73.1%)	219 (77.4%)	64 (22.6%)	1.391
Madhesi	24 (6.2%)	18 (75.0%)	6 (25.0%)	(0.851^a^)
Adiwasi/Janjati	69 (17.8%)	51 (73.9%)	18 (26.1%)	—
Dalit	3 (0.8%)	2 (66.7%)	1 (33.3%)	—
Other	8 (2.1%)	7 (87.5%)	1 (12.5%)	—
Current living residence				
Hostel	39 (10.1%)	30 (76.9%)	9 (23.1%)	10.493
With family	135 (34.9%)	96 (71.1%)	39 (28.9%)	(0.033)
Alone	93 (24.0%)	67 (72.0%)	26 (28.0%)	—
With friends	84 (21.7%)	74 (88.1%)	10 (11.9%)	—
With relatives	36 (9.3%)	30 (83.3%)	6 (16.7%)	—
Marital status				
Single	330 (85.3%)	258 (78.2%)	72 (21.8%)	4.904
Married	48 (12.4%)	34 (70.8%)	14 (29.2%)	(0.156^a^)
Divorced	1 (0.3%)	1 (100.0%)	0	—
Living together	8 (2.1%)	4 (50.0%)	4 (50.0%)	—
Family monthly income				
≤25 K	104 (26.9%)	75 (72.1%)	29 (27.9%)	3.396
Between 25 and 50 k	174 (45%)	132 (75.9%)	42 (24.1%)	(0.183)
≥50 k	109 (28.2%)	90 (82.6%)	19 (17.4%)	—
Financial stress				
No	163 (42.1%)	141 (86.5%)	22 (13.5%)	15.027
Yes	224 (57.9%)	1566 (69.6%)	68 (30.4%)	(0.001)
Choice of study				
Family pressure	21 (5.4%)	13 (61.9%)	8 (38.1%)	2.740
Self‐preference	366 (94.6%)	284 (77.6%)	82 (22.4%)	(0.098)
Parental relationship				
Poor	14 (3.60%)	9 (64.30%)	5 (35.70%)	4.868
Fair	70 (18.10%)	48 (68.60%)	22 (31.40%)	(0.088)
Good	303 (78.30%)	240 (79.20%)	63 (20.80%)	—
Chronic disease in the family				
No	324 (83.7%)	225 (78.7%)	69 (21.3%)	4.282
Yes	63 (16.3%)	42 (66.7%)	21 (33.3%)	(0.039)

*Note:* Pearson chi‐square test for categorical variables.

^a^Fisher’s exact test for categorical variables.

^b^ *t*‐test for numerical variables.

**Table 2:** Associations of personal and behavioral variables with depressive symptoms.

Variables	*n* (%)	Depressive symptoms		(*p*‐Value)
		No/low	Moderate/severe	
Smoking habits				
Never	324 (83.70%)	250 (77.20%)	74 (22.80%)	—
Present	13 (3.40%)	10 (76.90%)	3 (23.10%)	3.243
Past	14 (3.60%)	8 (57.10%)	6 (42.90%)	(0.349 ^∗^)
Occasional	36 (9.30%)	29 (80.60%)	7 (19.40%)	—
Alcohol consumption habits				
Never	231 (59.7%)	180 (77.9%)	51 (22.1%)	—
Present	16 (4.1%)	10 (62.5%)	6 (37.5%)	3.291
Past	14 (3.6%)	9 (64.3%)	5 (35.7%)	(0.349)
Occasional	126 (32.6%)	98 (77.8%)	28 (22.2%)	—
Death of a family member				
No	314 (81.1%)	249 (79.3%)	65 (20.7%)	6.090
Yes	73 (18.9%)	48 (65.8%)	25 (34.2%)	(0.014)
Relationship dissolution				
No	329 (85.0%)	259 (78.7%)	70 (21.3%)	4.818
Yes	58 (15.0%)	38 (65.5%)	20 (34.5%)	(0.028)
Worried about the future				
Not/slightly worried	115 (29.7%)	100 (87.0%)	5 (13.0%)	—
Moderately worried	114 (29.5%)	97 (85.1%)	17 (14.9%)	29.138
Very extremely/extremely worried	158 (40.8%)	100 (63.3%)	29 (36.7%)	(0.001)
Sleeping hours				
≤6 h	59 (15.2%)	41(69.5%)	18 (30.5%)	2.127
Between 6 and 8 h	265 (68.5%)	206 (77.7%)	59 (22.3%)	(0.345)
≥8 h	63 (16.3%)	50 (79.4%)	13 (20.6%)	—

*Note:* Pearson chi‐square test for categorical variables.

^∗^Fisher’s exact test for categorical variables.

**Table 3:** Associations of academic and psychological well‐being variables with depressive symptoms.

Variables	*n* (%)	Depressive symptoms		(*p*‐Value)
		No/low	Moderate/severe	
Manageable course load				
Disagree	81 (20.9%)	54 (66.7%)	27 (33.3%)	6.016
Neutral	138 (35.7%)	108 (78.3%)	30 (21.7%)	(0.049)
Agree	168 (43.4%)	135 (80.4%)	33 (19.6%)	—
Adequate academic support				
Disagree	78 (20.2%)	54 (69.2%)	24 (30.8%)	6.256
Neutral	102 (26.4%)	74 (72.5%)	28 (27.5%)	(0.044)
Agree	207 (53.5%)	169 (81.6%)	38 (18.4%)	—
Enjoyable lab work				
Disagree	55 (14.2%)	37 (67.3%)	18 (32.7%)	3.315
Neutral	92 (23.8%)	71 (77.2%)	21 (22.8%)	(0.191)
Agree	240 (62%)	189 (78.8%)	51 (21.3%)	—
Stressful assessments				
Disagree	114 (29.5%)	103 (90.4%)	11 (9.6%)	36.652
Neutral	119 (30.8%)	100 (84.0%)	19 (16.0%)	(0.001)
Agree	154 (39.8%)	94 (61.0%)	60 (39.0%)	—
Self‐esteem				
Low self‐esteem	58 (15.0%)	26 (44.8%)	18 (55.2%)	—
Medium self‐esteem	124 (32.0%)	82 (66.1%)	42 (33.9%)	68.354
High self‐esteem	205 (53.0%)	189 (92.2%)	16 (7.8%)	(0.001)
Perceived social support				
Weak support	135 (34.9%)	85 (63.0%)	50 (37.0%)	—
Moderate support	193 (49.9%)	160 (82.9%)	33 (17.1%)	22.755
Strong support	59 (15.2%)	52 (88.1%)	7 (11.9%)	(0.001)

*Note:* Pearson chi‐square test for categorical variables.

However, age, ethnicity, marital status, family income, choice of study, parental relationship, chronic disease in the family, smoking habits, alcohol consumption, sleeping hours, enjoyment in lab work, and positive relationships with subject‐specific teachers were not significant at the 5% level of significance. Therefore, by ignoring the independent variables that did not seem to be significant, a multivariable logistic regression model was constructed.

### 3.2. Multivariable Logistic Regression Model

A multivariable logistic regression model was used to investigate the associations between depressive symptoms and several independent variables that were significant at the 5% level in the bivariate analysis. The variables in the final model were selected using the forward selection procedure, where only four variables remained significant: financial stress (odds ratio [OR]: 2.35, 95% CI: 1.30–4.26), relationship dissolution (OR: 2.22, 95% CI: 1.06–4.63), having stressful assignments (OR: 5.09, 95% CI:2.31−11.26), and having medium self‐esteem (OR: 4.73, 95% CI: 2.43–9.18) and low self‐esteem (OR: 10.21, 95% CI: 4.71–22.11). The remaining variables initially considered, including gender, living arrangements, chronic illness in the family, death of a family member, worries about the future, course load, academic support, academic pressure, and PSS, did not remain significant in the final model. The results of the final multivariable logistic regression model are presented in Table 4.

**Table 4:** Result of multivariable logistic regression for depressive symptoms (*n* = 387).

Variables	*B*	SE	Wald	*p*‐Value	Odds ratio exp (*β*)	95% CI for odds ratio	
						Lower	Upper
Financial stress							
No	—	—	—	—	—	—	—
Yes	0.86	0.30	7.91	0.01	2.35	1.30	4.26
Relationship dissolution							
No	—	—	—	—	—	—	—
Yes	0.80	0.38	4.46	0.04	2.22	1.06	4.63
Stressful assessments							
Disagree	—	—	—	—	—	—	—
Neutral	0.46	0.45	1.06	0.30	1.58	0.66	3.81
Agree	1.63	0.40	16.20	0.01	5.09	2.31	11.26
Self‐esteem							
High self‐esteem	—	—	—	—	—	—	—
Medium self‐esteem	1.55	0.34	20.97	0.01	4.73	2.43	9.18
Low self‐esteem	2.32	0.39	34.67	0.01	10.21	4.71	22.11
Constant	−3.92	0.47	68.72	<0.001	0.02	—	—


*Note*: Log likelihood (only with intercept) = ‐ 419.78; log likelihood (full model) = −316.31. Nagelkerke *R* ^2^ = 0.354. Hosmer–Lemeshow *χ* ^2^ test with 8 df = 6.382, *p* = 0.605. AUC = 82.5% CI (77.4%, 87.6%).

**Figure 1:**
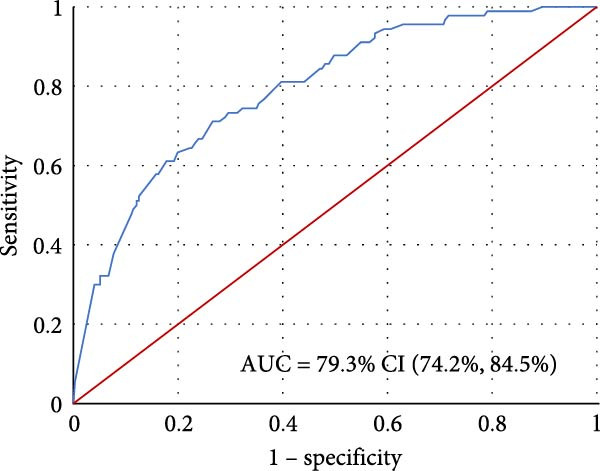
ROC curve for the model of depressive symptoms.

Our analysis yields a Nagelkerke *R*‐square of 0.354, indicating that the model explains ~35.4% of the variance in depressive symptoms among graduate students. Additionally, the Hosmer–Lemeshow test results (*χ* ^2^ = 6.38, *p* = 0.605) suggest a good fit for the model, with no significant difference between the observed and expected values between the respondents with no/low depressive symptoms and those with moderate/severe depressive symptoms. This is further supported by the ROC curve analysis, which yielded an AUC of 82.5%, indicating good discriminative capacity [19] with a 95% CI of 77.4% to 87.6% as shown in Figure 1.

## 4. Discussion

In our study, about one in four suffered moderate/severe depressive symptoms among graduate students. In multivariable analysis, the significant factors associated with depressive symptoms included financial stress, relationship dissolution, stressful assignments, medium self‐esteem, and low self‐esteem.

Our findings align closely with the results of prior studies that have explored the mental health of university students. Studies conducted among undergraduate students in Nepal show a depression rate of 20% in Gandaki Province [20], 21.2% in Chitwan and Kathmandu [9], and 29.2% at KIST Medical College [8] using the PHQ‐9 tool. Similarly, international studies have reported comparable findings, with depression rates of 26.8% among students in Ethiopia and Saudi Arabia [7, 21]. Furthermore, a meta‐analysis encompassing 27,717 postgraduate students over four decades estimated an overall pooled prevalence of 34%, with individual studies reporting rates ranging from 6.2% to 84.7% [3].

Among the identified risk factors, financial stress was found to be one of the major contributors to depressive symptoms, which is in line with earlier studies that show how it affected graduate students’ academic performance and well‐being [5, 22]. In Nepal, graduate students face stress due to the lack of part‐time job opportunities, rising urban living expenses, and the few scholarships for them [23–25]. This financial stress can lead to poor coping methods, such as a decrease in study time, sleep problems, and social isolation, which affect students’ academic progress and diminish their quality of life [26]. These findings suggest the need for universities to offer flexible payment methods, more scholarship opportunities, financial literacy campaigns, and easily accessible mental health services. Also, addressing financial stress can diminish mental distress, support academic progress, and enhance the well‐being of graduate students [27, 28].

Similarly, students who experience relationship dissolution are more likely to show depressive symptoms, which aligns with previous research conducted in Turkey [5] showing that master’s students in their 20s without romantic relationships were at higher risk of depression. The dissolution of such a relationship can cause sadness, loss of motivation, and anxiety due to the loss of emotional closeness and supportive relationships. Students with existing attachment insecurities may experience more depressive symptoms after a relationship ends due to ineffective coping methods, like self‐blame or low emotional adjustment [29]. Longitudinal studies also show that depressive symptoms increase right after a breakup but gradually decrease over time, depending on how individuals cope and handle the situation [30]. These findings highlight the need for emotional support and targeted interventions to help graduate students manage the psychological impact of romantic relationship losses.

Additionally, stressful assignments were found to be associated with depressive symptoms, consistent with findings from previous studies [31]. In Nepal, students may experience such stress due to demanding coursework and high workload, as well as a lack of mentoring and conventional teaching and assessment practices that emphasize memorization over creative thinking [32, 33]. Academic stress often leads to anxiety and feelings of hopelessness, which can increase the risk of depression [31]. These findings highlight the need for universities to rethink current curriculum design, improve academic mentoring, and provide accessible psychological support to manage academic stress and protect the mental well‐being of graduate students [31, 34].

Our finding also highlights the role of self‐esteem in mental health. Graduate students with medium and low self‐esteem were more likely to experience depressive symptoms, aligning with previous research that demonstrates the detrimental effects of low self‐esteem on mental health [35, 36]. In Nepal, societal pressures for academic excellence and a lack of recognition of individual achievements may lower graduate students’ self‐esteem. Such low self‐esteem can lead to negative self‐perceptions, self‐blame, and a reduced sense of control, which in turn increases feelings of hopelessness and social isolation [36, 37]. Therefore, for minimizing depressive symptoms and improving the mental well‐being of graduate students, universities should implement interventions that strengthen self‐esteem, such as peer support programs and skills‐based workshops that enhance confidence. Future research should continue to develop interventions to increase self‐esteem as a preventive measure against depressive symptoms in vulnerable graduate students. Future research should continue to develop interventions to increase self‐esteem as a preventive measure against depressive symptoms in vulnerable graduate students.

Several factors, including gender, current living residence, chronic disease in the family, family member death, manageable course load, adequate academic support, PSS, and worries about the future, showed associations with depressive symptoms in bivariate analysis but did not remain statistically significant in the multivariable logistic regression model after adjusting for other factors. While these factors might have contextual relevance and potentially influence depressive symptoms, further investigation is needed to determine their relationships with mental health outcomes.

Our study has several strengths and limitations. First, the cross‐sectional design prevents establishing causal relationships between depressive symptoms and associated factors. However, this design was chosen for its feasibility in assessing the prevalence and associated factors within a relatively short time frame. Second, the use of self‐reported data may introduce recall bias and inaccuracies in participants’ responses. Third, since the study was conducted in a single university, the results may limit generalizability to other universities in Nepal. However, these findings reflect common academic and psychosocial challenges faced by graduate students in similar higher education settings. Finally, the precision of the PSS measurement decreased due to a relatively low Cronbach’s alpha for the OSSS‐3 (0.582). Despite these limitations, the study has several strengths. It used validated tools, such as the PHQ‐9, to align with international research standards. It also provides an important baseline for educators, policymakers, and health professionals to develop and support graduate student mental health programs in Nepal. Conducting future studies using longitudinal designs and multi‐institutional samples would strengthen the evidence and provide more effective guidance for developing targeted interventions.

## 5. Conclusions

In summary, this study revealed that about one‐fourth (23%) of graduate students exhibited depressive symptoms. Factors such as financial stress, relationship dissolution, stressful assignments, and self‐esteem emerged as significant contributors to depressive symptoms within this specific student population. These results highlight the need for targeted interventions, such as accessible counseling services, peer‐support programs, and academic reforms that align teaching methods with students’ learning needs. Future research should examine these factors longitudinally and evaluate the effectiveness of such interventions. Universities should integrate mental health services into academic systems and make programs that address the challenges faced by graduate students in Nepal.

**Nomenclature**
IOST: Institute of Science and Technology
MSc: Master of science
OR: Odds ratio
OSSS‐3: Oslo Social Support Scale‐3
PHQ‐9: Patient Health Questionnaire‐9
ROC: Receiver operating characteristic
RSSE: Rosenberg self‐esteem scale
PSS: Perceived social support
SPSS: Statistical Package for Social Science
TU: Tribhuvan University
CI: Confidence interval
SE: Standard error
WHO: World Health Organization.

## Ethics Statement

This study was approved by the Institutional Review Committee of the Institute of Science and Technology, Tribhuvan University (Regd. No. IRCIOST‐24‐0001, approved title “Factors associated with depressive symptoms among graduate students of IOST, T.U., Nepal: a cross‐sectional study.” Written informed consent was obtained from all the participants in this study. The data obtained were kept confidential and anonymous to protect their privacy.

## Consent

Written informed consent was obtained from all the participants in this study.

## Disclosure

All the authors approved the manuscript.

## Conflicts of Interest

The authors declare no conflicts of interests.

## Author Contributions

Saphal Chapagai planned the study, collected and analyzed the data, and prepared the manuscript. Ishwari Prasad Banjade monitored the overall fieldwork and reviewed the manuscript. Shankar Prasad Khanal reviewed and edited the entire manuscript and provided feedback for the data analysis.

## Funding

This research was supported by the Research Directorate, Office of Rector, Tribhuvan University (Grant Number EF080‐81‐MT‐37).

## Data Availability

The data that support the findings of this study are available upon request from the corresponding author.
