# Correction to “Factors Associated With Depressive Symptoms Among Graduate Students in Science and Technology: A Cross‐Sectional Study With Multivariable Analysis”

**DOI:** 10.1155/da/9786494

**Published:** 2026-02-22

S. Chapagai, I. P. Banjade, and S. P. Khanal, “Factors Associated With Depressive Symptoms Among Graduate Students in Science and Technology: A Cross‐Sectional Study With Multivariable Analysis,” *Depression and Anxiety* 2025 (2025): 8886228, https://doi.org/10.1155/da/8886228.

In the article titled, “Factors Associated With Depressive Symptoms Among Graduate Students in Science and Technology: A Cross‐Sectional Study With Multivariable Analysis,” there was an error in the article type. The correct article type is Research Article.

We apologize for this error.

